# Progress of Dental Surgery

**Published:** 1842-06

**Authors:** 


*Progress of Dental Surgery.*—In a former number of our Journal we copied from *"The Boston Medical and Surgical Journal,"* a notice alluding to a communication that had appeared in the *"London Lancet,"* in which the efforts making in the country to advance the science of Dental Surgery, and elevate the respectability of the profession were favourably spoken of. The following, which we copy from the *"New York Lancet,"* is, we presume, the communication alluded to, and the editor in giving place to it, in connection with the "Second Annual Announcement of the Baltimore College of Dental Surgery," which we omit, says under the head of

*"Progress of Dental Surgery in the United States.*—We have in a former number of our journal, given a brief expression of the high gratification with which we, in common with all friends of science and of suffering humanity, witness the rapid strides which the art of dentistry is at present taking in its progress towards that elevated and honorable position to which it is justly entitled. We triumph in the advancement of surgery, and with an honest pride rejoice as we relate the evidences of its progress, and give permanent record to every fresh accession to its glory. And why should we discover less delight or enthusiasm, in observing the progress of another important branch of that noble profession, to which we have devoted our energies and talents? In this spirit, we cheerfully afford a considerable portion of our space to the following interesting articles—one illustrative of the present state and prospects of dental surgery in this country, and the other, expressive of the estimation in which the labors of our countrymen are held by enlightened minds in Europe.

PROGRESS OF DENTAL SCIENCE IN AMERICA.

*To the Editor of the London Lancet:*

SIR:—At a time like the present, when the subject of general medical reform occupies the especial attention of the profession, and the necessity of providing some protection for society against the inroads of empiricism, becomes daily more evident, it may not be out of place to direct attention to the strenuous efforts made on the other side of the Atlantic, to rescue a most useful, though perhaps humble branch of surgery, from the depths of degradation to which it has been reduced, by the disgraceful quackery of a great portion of those who profess to follow the avocations of the dental surgeon.

After encountering much opposition, and overcoming many serious difficulties, a few of the leading dentists in New York, aided by some equally praiseworthy professional brethren in Philadelphia, Baltimore, and Boston, succeeded early in 1839, in forming an association, from which shortly after emanated the first number of a monthly journal, under the title of the "American Journal of Dental Science;" the avowed objects of the publication being to afford facilities for the diffusion of knowledge in dental theory and practice, and for that unreserved intercommunication of facts between the members of the profession which ever opposes the firmest obstacle to the growth of quackery, inasmuch as one of the strongest distinctive attributes of an empiric is, *to have,* or *to pretend to have, professional secrets.*

The first volume of this journal has been completed in twelve numbers, and it has been determined to publish the second volume in four quarterly numbers.

The contents of the first volume have been of an exceedingly interesting and useful character, reflecting the greatest credit on the discernment of the able editors, Dr. Harris, of Baltimore, and Dr. Parmly, of New York.

Amongst the earliest beneficial results of the publication of this journal, has been the complete organization of the "American Society of Dental Surgeons;' a society which, amongst many other laudable designs, professes its object to be the establishment of dental colleges in the various states of the Union, and ultimately the conferring of a degree in dental surgery on those properly-qualified candidates who may be desirous of obtaining such evidence of their theoretical and practical knowledge.

In November, 1840, the Baltimore College of Dental Surgery commenced its first session, under the guidance of four professors; viz. of dental physiology and pathology, of anatomy and physiology, of practical dentistry, and of special pathology and therapeutics; and this institution is now in full and successful operation. Considerable progress has also been made towards the formation of a similar establishment in New York.

If facts and observations connected with dental pathology may be deemed worthy of a place in the *Lancet*, I shall experience much pleasure in offering to your notice occasionally some reports of the proceedings of the American Association, in the hope that a little agitation of the subject may be instrumental in exciting the attention of those eminent members of the profession who are best qualified, by the position they occupy, to bring about an effectual reform in this, as well as other branches of surgery. I have the honor to be, sir, your humble servant, W. H. Lintott.

